# Clinical phenotype spectrum and prognostic analysis of *DNM1L-*related disorders: a single-center cohort study of 18 patients

**DOI:** 10.3389/fneur.2026.1791244

**Published:** 2026-08-19

**Authors:** Han Xu, Chaolong Xu, Ying Zou, Xin Duan, Minhan Song, Xiaodi Han, Tianyu Song, Yang Liu, Fang Fang

**Affiliations:** Department of Neurology, Beijing Children's Hospital, Capital Medical University, National Center for Children's Health, Beijing, China

**Keywords:** *DNM1L* variant, encephalopathy due to defective mitochondrial and peroxisomal fission-1, genotype and phenotype, hemiconvulsion-hemiplegia-epilepsy syndrome, mitochondrial dynamics

## Abstract

**Aims:**

To summarize the clinical and genetic characteristics of *DNM1L*-related disorders and explore genotype-phenotype correlations and prognosis.

**Methods:**

We retrospectively analyzed clinical data from 18 children with *DNM1L* variants diagnosed between 2015 and October 2025. Combined with systematic literature review (2007-October 2025) to analyze reported *DNM1L* variant types and clinical phenotypes.

**Results:**

The cohort included 18 children (10 male, 8 female) with a median onset age of 3.5 years. Epilepsy occurred in 88.9% of patients, with 72.2% developing super-refractory status epilepticus; 66.7% had dystonia. Most patients exhibited brain MRI and EEG abnormalities. Eight novel pathogenic variants were identified (four missense, three frameshift, one compound heterozygous). Notably, eight patients with middle domain variants (primarily p.Arg403Cys) presented a novel “hemiconvulsion-hemiplegia-epilepsy syndrome” phenotype. At last follow-up, 83.3% had a modified Rankin Scale score ≥4, indicating severe disability and poor prognosis. Literatures review confirmed *DNM1L* variants are predominantly missense, with the middle domain as a hotspot. The p.Arg403Cys variant is recurrent and highly prevalent in the Chinese. Middle domain variants are more common in Asians, while GTPase domain variants are more frequent in Europeans. Missense variants in the middle domain correlated with higher rates of neurological dysfunction and mortality.

**Conclusions:**

This study represents an extension of our earlier work by incorporating an additional 18 cases, which expands the genetic and phenotypic spectrum of *DNM1L*-related disorders. It identifies “hemiconvulsion-hemiplegia-epilepsy syndrome” as a distinct feature and potential prognostic indicator for middle domain variants. The established genotype-phenotype patterns, based on protein domains and ethnic differences, provide critical insights for precise diagnosis and management.

## 1 Introduction

Mitochondria are highly dynamic organelles that maintain a relative balance between fusion and fission under physiological conditions to preserve cellular metabolic homeostasis-a process known as mitochondrial dynamics. Mitochondrial dynamics play a critical role in regulating various cellular functions, including mitochondrial biogenesis, autophagy, apoptosis, interactions with other organelles (such as the endoplasmic reticulum), and signal transduction. Disruption of mitochondrial dynamics-imbalance between fission and fusion-compromises mitochondrial integrity and contributes to a range of mitochondrial diseases and neurodegenerative diseases, including Alzheimer's, Parkinson's, and Huntington's diseases (1). Mitochondrial fusion is mediated by mitofusin 1 and 2 (MFN1/2) and optic atrophy 1 (OPA1), while fission is primarily regulated by dynamin-related protein 1 (DRP1), mitochondrial fission protein 1 (FIS1), and mitochondrial fission factor (MFF). Moreover, the endoplasmic reticulum (ER) also participates in mitochondrial fission in two ways: (i) critical contact between ER tubules and mitochondria occurs at specific sites, which warrants midzone fission for mitochondrial proliferation (2), and (ii) peripheral contact warrants peripheral fission and the clearance of damaged mitochondria (3).

DRP1, a GTPase encoded by the *DNM1L* gene on chromosome 12, belongs to the dynamin superfamily and regulates mitochondrial and peroxisomal fission. It is widely expressed across various tissues (4). DRP1 is essential for normal embryonic development; constitutive homozygous knockout of DRP1in mice leads to embryonic lethality, while conditional knockout results in developmental defects associated with impaired mitochondrial fission. Neurons are particularly sensitive to DRP1 dysfunction. Studies have shown that conditional *DNM1L* knockout mice exhibit significant hypoplasia of the white matter and cerebellum, as well as morphologically abnormal giant mitochondria in basal ganglia neurons and Purkinje cells, underscoring the critical role of DRP1 in central nervous system development (5).

The mechanisms linking DRP1 dysfunction to epilepsy remain poorly understood. Maria et al. (6) suggested that abnormal DRP1-mediated mitochondrial fission may disrupt cortical development and promote epileptogenesis by altering astrocyte morphology and organization. Lee et al. (7) reported that in a chronic epilepsy rat model, hyperphosphorylation of DRP1 at S637—mediated by the HSP25-AKT pathway—led to abnormal mitochondrial elongation, triggering autophagic degeneration of astrocytes and contributing to seizure generation. Zhang et al. (8) found that Rho GTPase-activating protein 44 (RICH2) upregulates DRP1 expression, inhibiting mitochondrial fusion and leading to the release of dysfunctional mitochondria into the extracellular environment. These mitochondria are subsequently taken up by neurons, promoting epileptogenesis in diffuse low-grade gliomas.

The DRP1 protein consists of four domains: the GTPase domain (GD), middle domain (MD), variable domain (VD), and GTPase effector domain (GED) (2). Each domain plays distinct yet interconnected roles in DRP1 function. However, comprehensive genotype-phenotype correlation studies are still lacking. Encephalopathy due to defective mitochondrial and peroxisomal fission-1 (EMPF1) is a rare metabolic encephalopathy caused by *DNM1L* mutations. These mutations lead to defective DRP1 function, resulting in impaired mitochondrial and peroxisomal fission and multisystem dysfunction with overlapping phenotypic features. In 2007, Waterham et al. (9) first reported a female infant with a de novo heterozygous pathogenic *DNM1L* variant who presented with lethal encephalopathy, featuring brain maldevelopment, frequent seizures, lactic acidosis, elevated very-long-chain fatty acids, and elongated mitochondria and peroxisomes. The infant survived only 37 days. EMPF1 exhibits both clinical and genetic heterogeneity, with most cases following autosomal dominant inheritance (AD), though rare autosomal recessive (AR) inherited cases have been reported. Depending on the variant's location, type, and impact on mitochondrial and peroxisomal function, the age of onset, clinical manifestations, and disease severity vary widely—ranging from early lethal encephalopathy in infants to rare isolated optic atrophy, or even sudden-onset refractory status epilepticus in childhood following minor metabolic insults, progressing to fatal epileptic encephalopathy with diffuse cerebral atrophy and severe neurological disability. Some patients die in infancy or early childhood, reflecting an extremely poor prognosis. Additional features may include microcephaly, ataxia, nystagmus, peripheral neuropathy, and cardiac or digestive system abnormalities.

Refractory epilepsy with super-refractory status epilepticus (SRSE) is one of the most common neurological manifestations of EMPF1. SRSE is a severe condition defined as status epilepticus that persists beyond 24 h of anesthetic treatment or recurs upon tapering or withdrawal of anesthetics. It is resistant to conventional antiseizure medications (ASMs) and often requires complex multimodal therapy. *DNM1L* variants disrupt mitochondrial and peroxisomal fission, leading to dysregulated mitochondrial dynamics and energy metabolism. This results in mitochondrial dysfunction that fails to meet increasing cellular energy demands, causing diffuse neuronal and glial necrosis, progressive neurological decline, and ultimately SRSE.

Although multiple studies have reported various *DNM1L* variants and their clinical presentations, most focus on specific mutation types and are limited to case reports (9–38). There is a lack of systematic summaries correlating variant types with clinical phenotypes and comprehensive comparisons of outcomes across different mutations. This gap hinders precise diagnosis and limits exploration of potential treatments. Furthermore, the distinct molecular and clinical profiles of different *DNM1L* variants have precluded clear genotype-phenotype correlations. Thus, molecular diagnosis and phenotypic characterization of novel pathogenic *DNM1L* variants are crucial for elucidating the complexity of the mutational and phenotypic spectrum.

Our team previously reported 11 Chinese children with *DNM1L* variants, noting that those with MD mutations (particularly p.Arg403Cys) exhibited a unique electroencephalographic pattern termed rhythmic high-amplitude delta with superimposed polyspikes (RHADS). All affected individuals developed SRSE, suggesting that RHADS may serve as an early diagnostic marker and prognostic indicator of severity (39). Building upon our previously published cases, we have further expanded the cohort and included 18 newly diagnosed patients with *DNM1L* variants. We aim to summarize their clinical and genetic characteristics, broaden the genotypic and phenotypic spectrum, and analyze clinical outcomes associated with pathogenic variants in different DRP1 domains, as well as ethnic distribution patterns. Through detailed case analysis and literature review, we seek to enhance understanding of *DNM1L*-related disorders and facilitate early diagnosis and personalized treatment.

## 2 Materials and methods

### 2.1 Study subjects

The Department of Neurology at Beijing Children's Hospital, Capital Medical University, hosts China's largest single-center cohort of pediatric mitochondrial diseases. To date, 1,050 children with mitochondrial disorders have been molecularly diagnosed, including 524 cases (49.9%) with nuclear gene mutations and 526 cases (50.1%) with mitochondrial DNA variants. We retrospectively collected clinical data from children diagnosed with *DNM1L* variants at Beijing Children's Hospital between June 2015 and October 2025. Inclusion criteria were as follows: (i) Patients aged ≤ 18 years with complete clinical records, including follow-up data and deceased cases. (ii) Identification of a *DNM1L* variant through whole-exome or whole-genome sequencing, classified as pathogenic (P) or likely pathogenic (LP) according to the American College of Medical Genetics and Genomics (ACMG) guidelines (40), with clinical manifestations consistent with previously reported phenotypes of *DNM1L*-related disorders. (iii) Absence of other genetic or non-genetic causes highly correlated with the clinical presentation. Exclusion criteria included cases with compatible clinical phenotypes but lacking genetic confirmation or incomplete clinical documentation. Building upon our previously published 11 cases, we have further expanded the cohort by including an additional 18 patients.

This study has been approved by the Ethics Committee of Beijing Children's Hospital, Capital Medical University [Approval number: (2022)-E-121Y]. Furthermore, written informed consent forms have been signed by the parents or guardians of all participants, authorizing the use of data for research purposes.

### 2.2 Clinical data collection

Demographic and clinical information was summarized for all patients, including sex, age at onset, initial symptoms, seizure types, age at seizure onset, seizure frequency, number of ASMs used, genetic testing results, perinatal abnormalities, family history of epilepsy or other neurological disorders, electroencephalography (EEG) findings, neuropsychological assessments, neuroimaging results, and outcomes at last follow-up. Given that *DNM1L* variants may cause multi-system dysfunction, extra-neurological symptoms and auxiliary examination results were also documented. Drug-resistant epilepsy and status epilepticus were defined according to the International League Against Epilepsy (ILAE) consensus (41).

### 2.3 Genetic testing

Peripheral blood samples from all patients and their parents underwent trio whole-exome or whole-genome sequencing using the Illumina platform, with an average sequencing depth >100 × . Mitochondrial genome sequencing was simultaneously performed with an average depth >20,000 × . Sequencing data were aligned to the GRCh37/hg19 reference genome, and variants were filtered using the Ingenuity system. Common variants (frequency >1% in population databases such as 1000 Genomes, ExAC, and gnomAD) and non-functional variants (e.g., non-coding region changes) were excluded. Candidate variants were selected based on clinical symptoms, disease databases, and literature, followed by family validation. Pathogenicity assessments included *in silico* predictions of protein impact (using PROVEAN, SIFT, PolyPhen-2, MutationTaster, and REVEL) and splice-altering effects (using MaxEntScan, dbscSNV, and GTAG). Final pathogenicity was determined according to ACMG guidelines. Data on gene name, transcript, mutation type and position, amino acid change, variant origin, and inheritance pattern were systematically collected.

### 2.4 Literature search strategy

We searched the China National Knowledge Infrastructure (CNKI), PubMed, Web of Science, and other databases for articles published between 2007 and October 2025 using the keywords “*DNM1L* gene,” “*DNM1L* variant,” or “*DNM1L* mutation.” Included studies reported pathogenic or likely pathogenic *DNM1L* variants confirmed by genetic testing, with clear clinical phenotypic data. Case reports, case series, and relevant academic theses were included, while duplicate reports, animal studies, and basic research articles were excluded.

### 2.5 Statistical analysis

Categorical variables were summarized as counts and percentages, and continuous variables as medians and ranges. All statistical analyses and data visualization were performed using GraphPad Prism v9 and SPSS 26.0. No outliers were removed during analysis.

## 3 Results

### 3.1 Demographic and clinical characteristics

A total of 18 children with *DNM1L* variants were enrolled in this study. Their demographic and clinical characteristics are summarized in  Supplementary Table S1. Two pedigrees each had two male probands: one pair consisted of monozygotic twins (Cases 10 and 11) carrying a de novo heterozygous missense variant; the other pair were siblings (Cases 9 and 18) with compound heterozygous variants inherited from phenotypically normal parents. None of the children had perinatal abnormalities or a family history of neurological disorders such as epilepsy. The male-to-female ratio was 5:4 (10 males, 8 females). The main clinical manifestations were drug-resistant epilepsy and global developmental delay, affecting over 90% of the patients. The median age of onset was 3.5 years (range: 0.1–10.8 years). The initial symptoms included developmental delay (33.3%, 6/18) and epileptic seizures (44.4%, 8/18), with seizure types encompassing generalized tonic-clonic, myoclonic, clonic, spasms, and focal seizures. Other initial presentations included muscle weakness, autism spectrum disorders (ASDs), feeding difficulties, and cyclic vomiting.

Epilepsy occurred in 88.9% (16/18) of the children during the disease course, with a median seizure onset age of 5.0 years (range: 0.2–11.5 years). All cases were refractory epilepsy. Seizure types included focal seizures (*N* = 12), generalized seizures (*N* = 1), and a combination of both (*N* = 3). Most experienced daily seizures, while a few had several episodes per year. Fifteen patients received ASMs, with an average of 6 different ASMs used (range: 1–8). SRSE occurred in 72.2% (13/18) of the children. In addition to conventional ASMs, these patients received anesthetic sedation (12/13) and other combination therapies, including cocktail therapy (12/13), corticosteroids (8/13), intravenous immunoglobulin (IVIG, 4/13), tocilizumab (3/13), and bezafibrate (2/13), with limited efficacy. Notably, probands with initial developmental delay often developed epilepsy years later, suggesting that refractory epilepsy and SRSE may be common outcomes in these patients.

All children underwent neuropsychological assessments, with 61.1% showing mild to moderate developmental delays. Five cases had normal or mildly delayed development before onset but experienced developmental regression after disease onset, partially associated with triggers such as fever, infection, vaccination, or status epilepticus. Dystonia was also common (66.7%, 12/18). A minority had nystagmus, optic atrophy, ataxia, spasticity, hemiplegia, muscle atrophy, or peripheral neuropathy, which worsened under stress and severely impacted daily life and care.

**Figure 1:**
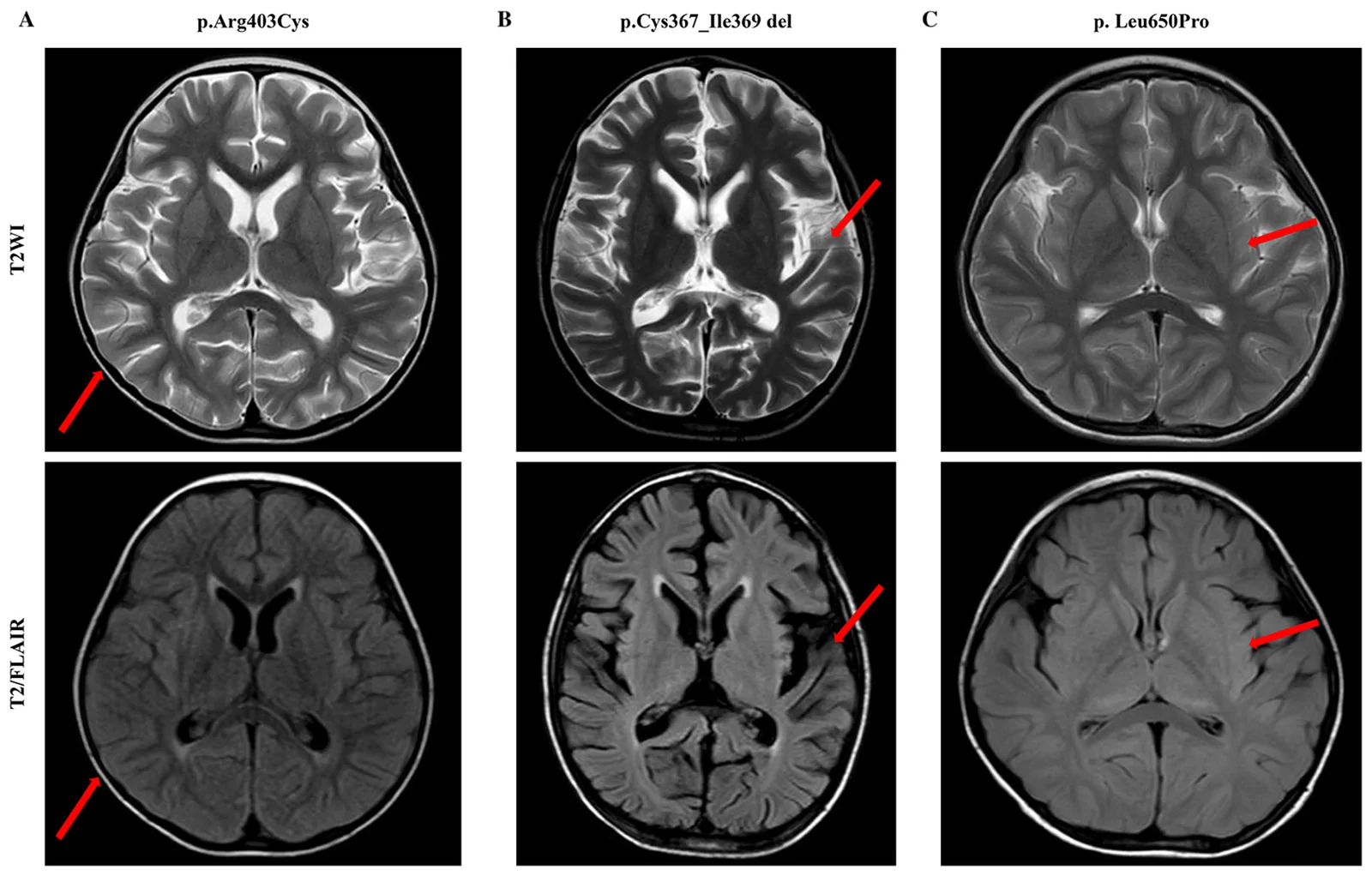
Representative axial brain MRI images of 3 patients with different *DNM1L* variants. **(A)** 4y7m, girl, 1 month since onset. The left column shows extensive swelling of the cerebral cortex in both hemispheres with hyperintensity on T2/FLAIR sequences. **(B)** 4y6m, boy, 1 year after onset. The middle column shows global cerebral atrophy and cerebral softening with gliosis. **(C)** 7y1m, boy, duration of illness: 2 weeks. The right column shows multiple T2 and T2/FLAIR hyperintensities in the bilateral cerebral cortex, along with symmetric lesions in the bilateral basal ganglia and thalamus. The red arrowhead indicates the representative area of the lesion. T2WI, T2-weighted image; T2/FLAIR, T2-Fluid-attenuated inversion-recovery sequency.

EEG revealed circumscribed discharges in 27.8% (5/18), widespread discharges in 23.2% (4/18), and bilateral independent or multifocal discharges in 50.0% (9/18) patients. Among the 16 children who underwent brain magnetic resonance imaging (MRI), 81.3% (13/16) showed abnormalities, often manifesting as acute unilateral or bilateral hemispheric diffusion restrictions with T2 fluid-attenuated inversion recovery sequence (T2/FLAIR) hyperintensity and cortical edema, involving the basal ganglia, thalamus, and hippocampus. Follow-up imaging revealed diffuse global cerebral atrophy with ventricular enlargement (Figure 1). A few cases showed only atypical tuberous sclerosis or delayed myelination (Supplementary Table S3). Three cases exhibited lactate peaks on magnetic resonance spectroscopy (MRS). Lactic acidosis was present in 16.7% (3/18). Two cases showed elevated serum or cerebrospinal fluid interleukin-6 (IL-6) during acute encephalopathy, with symptom improvement after tocilizumab treatment, suggesting neuroinflammation may contribute to disease progression.

Beyond neurological symptoms, one case had severe cardiac involvement. Other manifestations included rickets, conjunctival hyperemia, posterior urethral obstruction, biliary atresia, joint contractures, strabismus, and astigmatism. Based on the modified Rankin Scale (mRS), 83.3% of the children had scores ≥4 at the last follow-up, indicating high disability rates and poor prognosis. Currently, there are no effective treatments for *DNM1L*-related necrotizing encephalopathy.

### 3.2 Genetic results and genotype-phenotype correlation

**Figure 2:**
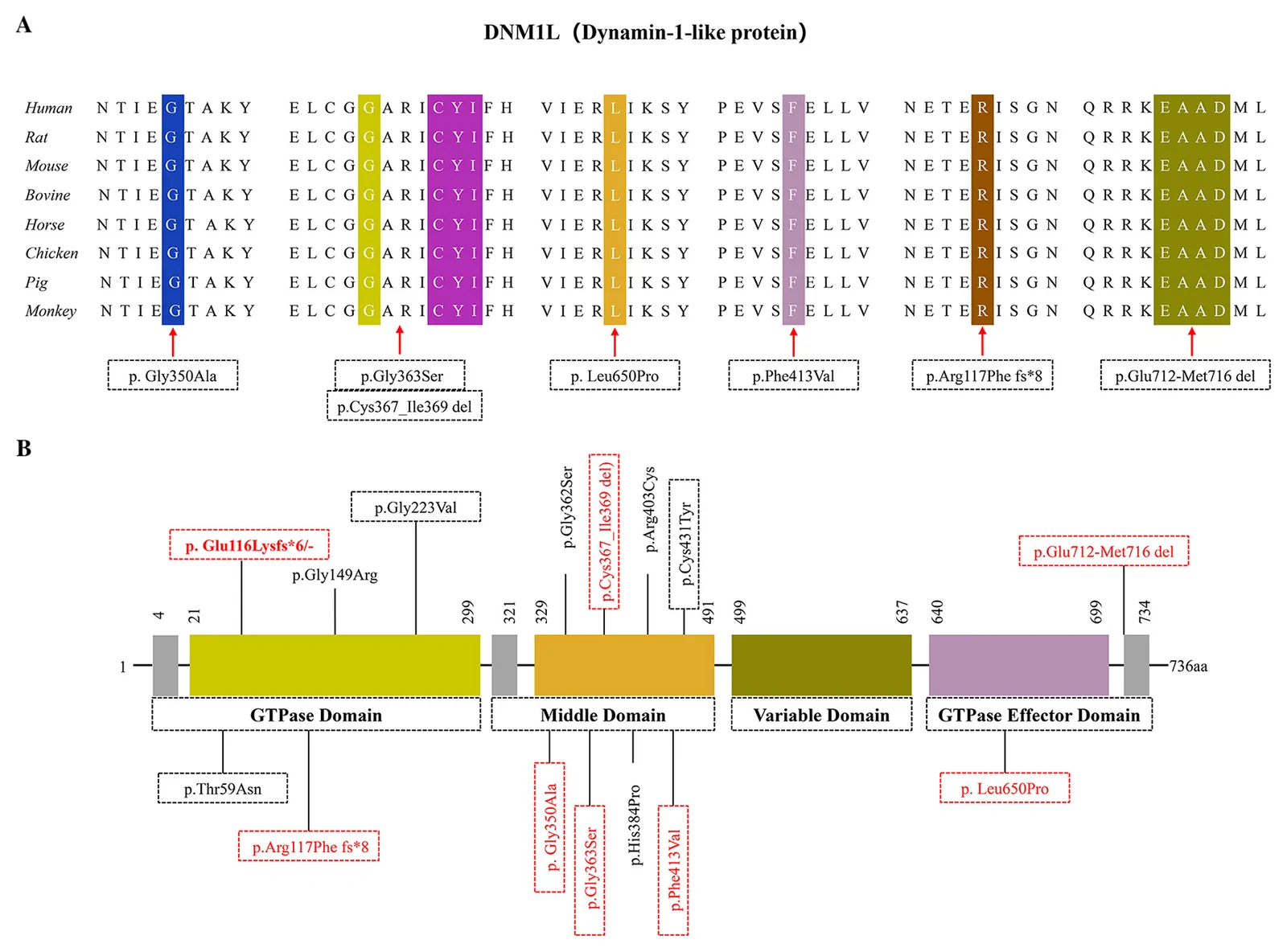
Distribution of *DNM1L* variants and Inter-species homologous conservation. **(A)** Analysis of homologous conserved sequences among species (7 representative novel variants). The red arrow indicates the mutated amino acid. **(B)** Schematic secondary structure of the *DNM1L* protein and the distribution of all variants in our cohort. Eight of these variants were newly identified in this study. The red box indicates a new, previously unreported variant.

Among the 18 individuals, 16 had de novo heterozygous variants with AD inheritance, while two brothers from one family had compound heterozygous variants with AR inheritance. The amino acid changes caused by these variants were highly evolutionarily conserved (Figure 2A). Among AD cases, 81.3% (13/16) were missense variants, and 18.7% (3/16) were frameshift variants (including two deletions and one truncation). The most common variant was *DNM1L* c.1207C>T (p.Arg403Cys), accounting for 37.5% (6/16), suggesting it may be a hotspot mutation. In AR cases, one allele had a missense variant and the other an intronic variant. Additionally, we identified eight novel unreported variants: *DNM1L* c.1049G>C (p.Gly350Ala), c.1087G>A (p.Gly363Ser), c.1949T>C (p.Leu650Pro), c.1292G>A (p.Cys431Tyr), c.349_352del (p.Arg117Phe fs^*^8), c.2133_2147del (p.Glu712_Met716del), c.1100_1108del (p.Cys367_Ile369del), and a compound heterozygous variant *DNM1L* c.345_346del/c.1885-25A>C, including four missense, three frameshift, and one compound heterozygous variant (Table 1).

**Table 1:** Details of *DNM1L* variants present in our cohort.

No.	Gene	cDNA Change	Amino acid change	Novel	Variation type	Structural domain	inheritance pattern	Variation mode	ACMG	Cases
1	*DNM1L*	c.1207 C>T	p.Arg403Cys	N	Mis	MD (*N* = 11)	AD	*De novo*	P	6
2	*DNM1L*	c.1049 G>C	p.Gly350Ala	Y	Missense		AD	*De novo*	LP	1
3	*DNM1L*	c.1087 G>A	p.Gly363Ser	Y	Missense		AD	*De novo*	LP	1
4	*DNM1L*	c.1100_1108 del	p.Cys367_Ile369 del	Y	Frameshift		AD	*De novo*	LP	1
5	*DNM1L*	c.1237 T>G	p.Phe413Val	Y	Missense		AD	*De novo*	LP	1
6	*DNM1L*	c.1292 G>A	p.Cys431Tyr	N	Missense		AD	*De novo*	P	1
7	*DNM1L*	c.668 G>T	p.Gly223Val	N	Missen	GD (*N* = 3)	AD	*De novo*	LP	1
8	*DNM1L*	c.349_352 del	p.Arg117Phe fs^*^8	Y	Frameshift		AD	*De novo*	LP	1
9	*DNM1L*	c.176 C>A	p.Thr59Asn	N	Missense		AD	*De novo*	LP	1
10	*DNM1L*	c.2133_2147 del	p.Glu712-Met716 del	Y	Frameshif	GED (*N* = 2)	AD	*De novo*	LP	1
11	*DNM1L*	c.1949 T>C	p.Leu650Pro	Y	Missense		AD	*De novo*	LP	1
12	*DNM1L*	c.345_346del/c.1885-25A>C Intron 17/19	p.Glu116Lysfs^*^6/-	Y	Frameshift		AR	Paternal	P	2
					intronic mutation	GD		Maternal	LP	

Y, yes, the variant was not reported; N, no, the variant was reported; ACMG, American college of medical genetics; AD, autosomal dominant inheritance; P, pathogenic; LP, likely pathogenic; MD, middle domain; GD, GTPase domain; GED, GTPase effector domain; Y, Yes; N, no; EMPF1, encephalopathy, lethal, due to defective mitochondrial peroxisomal fission 1; ASD, autism spectrum disorders.

**Figure 3:**
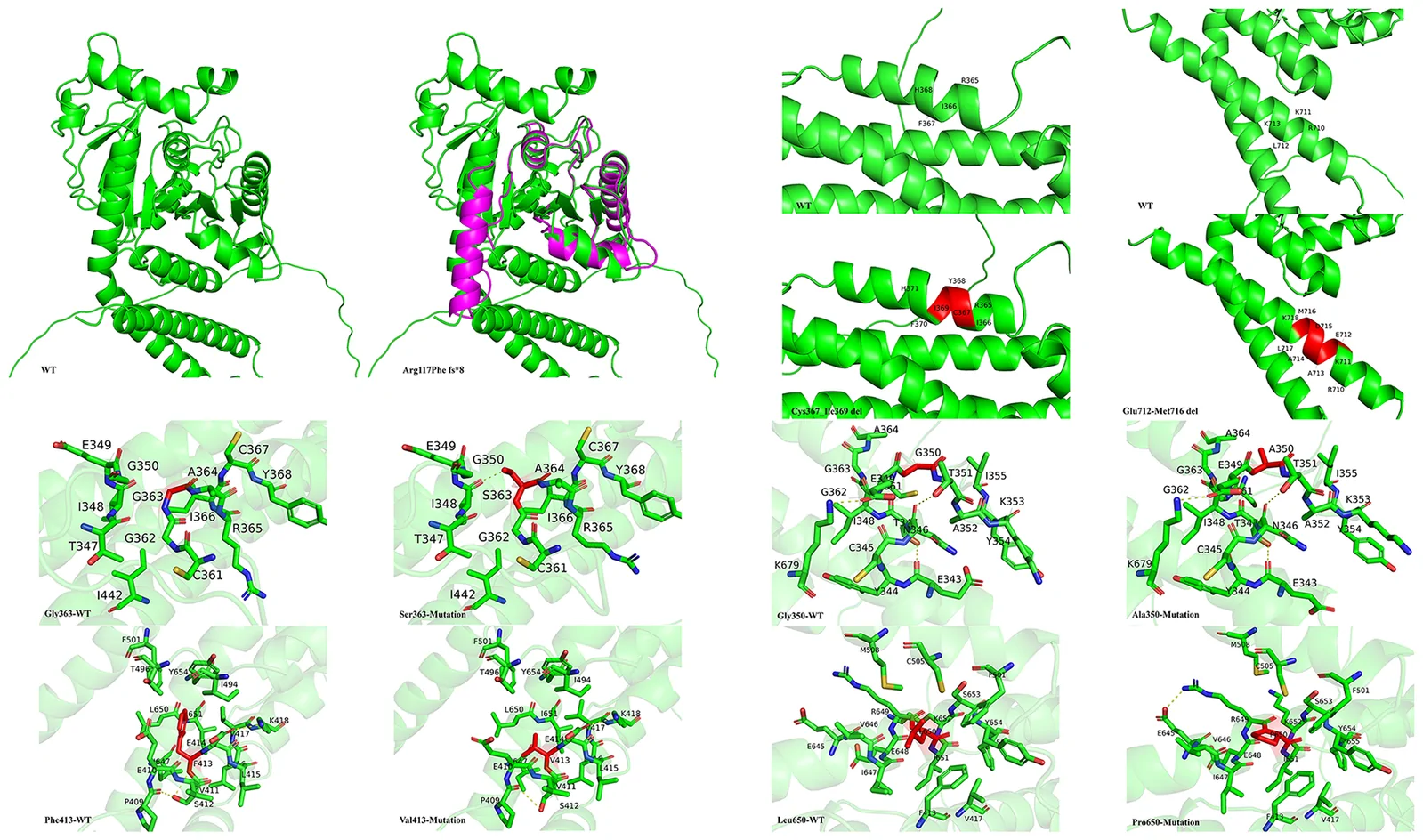
Distribution of *DNM1L* variants and Inter-species homologous conservation. Post-mutation amino acid structure alterations (7 representative unreported variants). Molecular structures were rendered with the PyMOL (http://www.pymol.org). Mutant sites are marked in purple and red. Missense mutations cause amino acid substitutions and alterations in hydrogen bond connections. Deletion mutations result in amino acid loss and truncating mutation lead to premature translation termination.

We mapped the locations of all *DNM1L* variants identified in this cohort to different domains of the DRP1 protein (Figure 2B). AD variants were located in the GD (16.7%), MD (61.1%, including the hotspot p.Arg403Cys), and GED (11.1%); another 11.1% were AR variants. Among patients with MD variants, 90.9% (10/11) exhibited typical EMPF1; those with GD and GED variants had diverse clinical presentations. Both children with AR variants showed typical EMPF1. One child with an MD missense variant (p.Cys431Tyr) died at the last follow-up. The three frameshift variants were evenly distributed across the three domains and were less common than missense variants. Homology modeling of the protein structure (Figure 3) revealed that MD variants may affect DRP1 oligomerization and downstream protein recruitment by altering hydrogen bonding, thereby impairing function and leading to more severe mitochondrial and peroxisomal fission defects.

Additionally, monozygotic twins carrying the same de novo variant (p.Arg403Cys) had different clinical presentations: one presented with developmental delay and underwent liver transplantation for biliary atresia, followed by long-term immunosuppressive therapy; the other presented with epilepsy. Both eventually developed refractory focal epilepsy and status epilepticus, highlighting significant phenotypic heterogeneity in this disease. No clear association was found between other variants and phenotypic severity, possibly due to the limited sample size and diverse clinical manifestations. Notably, 22.2% (4/18) of the patients developed hemiplegia after onset, presenting with unilateral clonic seizures and focal status epilepticus, which progressed to refractory focal epilepsy. The median age of onset for this group was 1.9 years (range: 0.3–5.8 years). Ictal EEG showed multifocal or generalized discharges with asymmetry; acute brain MRI revealed unilateral cortical edema and basal ganglia-thalamic abnormalities, progressing to diffuse cerebral atrophy, highly consistent with hemiconvulsion-hemiplegia-epilepsy syndrome (HHE). Three cases carried the p.Arg403Cys variant, and one carried p.Cys367_Ile369del, both located in the MD, suggesting that variants in this domain (especially p.Arg403Cys) may predispose to the novel HHE phenotype.

### 3.3 Literature review

A systematic literature search from 2007 to October 2025 identified 48 relevant publications (44 in English, 4 in Chinese). After excluding cases with incomplete clinical data or duplicate reports, 44 articles were included (9–38). To date, 44 distinct *DNM1L* variants have been reported across 107 patients of diverse ethnicities/nationalities (44 Asians, 45 Europeans, 17 North Americans, 1 South American). Among these, 40 variants followed AD inheritance (38 de novo heterozygous, 2 inherited), while 4 exhibited AR inheritance (from carrier parents). Among AD variants, missense variants accounted for 95.0% (95/100), frameshift for 4.0% (4/100), and nonsense for 1.0% (1/100). For AR variants, compound heterozygous constituted 85.7% (6/7), and homozygous 14.3% (1/7). The most frequent variant was *DNM1L* c.1207C>T (p.Arg403Cys), representing 30.8% (33/107) of all cases. Notably, 54.5% (18/33) of these were Chinese patients, suggesting this site may be a mutation hotspot in Asian populations, particularly among Chinese. The second most common site involved Gly362, with 7 reported cases (including p.Gly362Ser and p.Gly362Asp); all others were sporadic.

**Figure 4:**
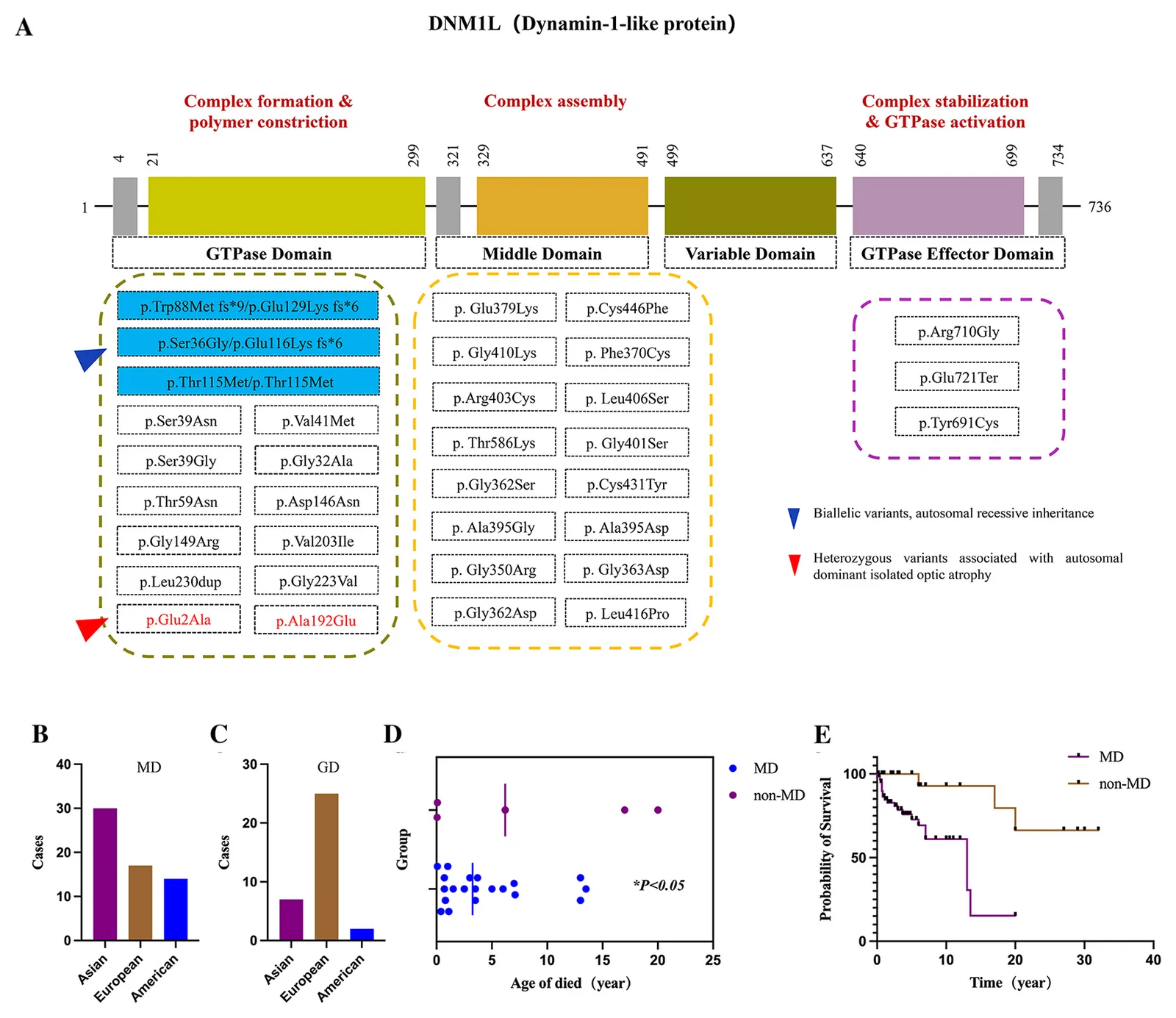
The *DNM1L* variant spectrum and genotype-phenotype correlation analysis. **(A)** *DNM1L* variants reported in the literature and their distribution across different domains. And functions of different domains. **(B, C)** Variation in different domains of *DNM1L* across ethnic groups. The middle domain variants show a higher prevalence of Asian, while the GTPase domain variants are more commonly observed in Europeans. **(D, E)** The age of died in MD group vs. non-MD group. Survival analysis between MD group and non-MD group. *P < 0.05.

Analysis of variant distribution across protein domains (Figure 4A) revealed that AD variants were predominantly located in the MD (57.0%, 61/107), GD (31.8%, 34/107), and GED (4.7%, 5/107). AR variants were exclusively found in the GD (6.5%, 7/107). Ethnic distribution analysis (Figures 4B, C) showed that 49.2% (30/61) of MD variants occurred in Asians (27 of whom were Chinese), while 73.5% (25/34) of GD variants were identified in Europeans. GED variants showed no significant ethnic predilection.

Combining data from this study and published cases (16 with isolated optic atrophy and incomplete records were excluded), clinical summaries were compiled for 91 patients (Table 2). Median age of onset was 3 years (range: 0–30 years). The most common manifestations were: global developmental delay/regression (87.9%, 80/91), seizures (72.5%, 66/91), dystonia (64.8%, 59/91), and status epilepticus (53.8%, 49/91). EMPF1 was observed in 74.7% (68/91). Other features included ataxia (18.7%), lactic acidosis (16.5%), microcephaly (13.2%), sensory/motor axonal neuropathy (12.1%), and nystagmus (10.9%). Mortality was 27.5% (25/91) at last follow-up. Brain MRI abnormalities were present in 78.0% (71/91); MRS showed elevated lactate peaks in 50.0% (6/12). EEG abnormalities occurred in 81.3% (74/91), commonly featuring diffuse background slow-wave activity (*N* = 13) and epileptiform discharges (*N* = 66). Previous work from our center indicated that RHADS was a distinctive EEG pattern in 9 patients carrying p.Arg403Cys variant (37). Among 30 patients who underwent muscle/skin biopsy, 11 showed reduced mitochondrial respiratory chain enzyme activity, and 22 had abnormal mitochondrial/peroxisomal morphology.

**Table 2:** The clinical characteristics in patients with *DNM1L* variants in different domains.

Clinical data %(*N*/*N*) or *N*	AD			AR (*N* = 7)	Total morbidity % (*N*/*N*)
	GD (*N* = 18)	MD (*N* = 61)	GED (*N* = 5)		
**Age of onset (y)**	1 (0–30)	3 (0–13)	1.5 (0–7.1)	1 (0–5)	3 (0–30)
Sex					
Male	27.8% (5/18)	47.5% (29/61)	60.0% (3/5)	71.4% (5/7)	46.2% (42/91)
Female	72.2% (13/18)	52.5% (32/61)	40.0% (2/5)	2.6% (2/7)	53.8% (49/91)
Follow-up outcome					
Alive	88.9% (16/18)	67.2% (41/61)	80.0% (4/5)	71.4% (5/7)	72.5% (66/91)
Died	11.1% (2/18)	32.8% (20/61)	20.0% (1/5)	28.6% (2/7)	27.5% (25/91)
**Modified Rankin scale (mRs)**	3 (3–6)	5 (3–6)	4 (3–6)	4 (4–6)	5 (3–6)
**Developmental delay or regression**	88.9% (16/18)	86.9% (53/61)	80.0% (4/5)	100% (7/7)	87.9% (80/91)
**Epilepsy**	33.3% (6/18)	90.2% (55/61)	60.0% (3/5)	28.6% (2/7)	72.5% (66/91)
Status epilepticus	22.2% (4/18)	66.7% (40/61)	60.0% (3/5)	28.6% (2/7)	53.8% (49/91)
**Dystonia**	77.8% (14/18)	60.7% (37/61)	40.0% (2/5)	85.7% (6/7)	64.8% (59/91)
**Peripheral neuropathy**	38.9% (7/18)	0	0	0	7.7% (7/91)
**Microcephaly**	22.2% (4/18)	13.1% (8/61)	0	0	13.2% (12/91)
**Encephalopathy**	11.1% (2/18)	96.7% (59/61)	40.0% (2/5)	71.4% (5/7)	74.7% (68/91)
**Ataxia**	50.0% (9/18)	9.8% (6/61)	0	28.6% (2/7)	18.7% (17/91)
**Nystagmus**	4	3	1	2	10.9% (10/91)
**Optic atrophy**	3	2	2	0	7.7% (7/91)
**Dysarthria**	4	3	0	4	12.1% (11/91)
**Pain insensitivity**	1	2	0	0	3.3% (3/91)
**Sensory and motor axonal neuropathy**	61.1% (11/18)	0	0	0	12.1% (11/91)
**Strabismus**	4	1	1	2	8.8% (8/91)
**Hyperlactacidemia**	0	21.3% (13/61)	0	28.6% (2/7)	16.5% (15/91)
**Abnormal brain MRI**	50.0% (9/18)	86.9% (53/61)	60.0% (3/5)	85.7% (6/7)	78.0% (71/91)
Lactate peak	0	5	0	1	50.0% (6/12)
**Abnormal EEG**				81.3% (74/91)	
Epileptiform discharge	33.3% (6/18)	96.7% (59/61)	60.0% (3/5)	28.6% (2/7)	76.9% (70/91)
Weak background	3	9	1	0	14.3% (13/91)
**Decreased respiratory chain enzyme activity**	1	9	0	1	
**Abnormal morphology of mitochondria**	6	12	1	3	

N, number; AD, autosomal dominant inheritance; AR, autosomal recessive inheritance; MD, middle domain; GD, GTPase domain; GED, GTPase effector domain; y, years; mRs, modified Rankin scale; MRI, magnetic resonance imaging; EEG, electroencephalography.

This is an exploratory analysis due to the limited sample size. Phenotypic analysis was conducted based on inheritance pattern and domain location revealed:

### 3.4 AD inheritance—MD

Median onset age: 3 years (range: 0–13 years). Drug-resistant epilepsy occurred in 90.2%, with 66.7% progressing to status epilepticus. This group showed significantly higher rates of refractory epilepsy, status epilepticus, and encephalopathy compared to others, but lower incidence of peripheral neuropathy and ataxia. Diffuse progressive cerebral atrophy was observed on MRI in 86.9%. Lactic acidosis was present in 21.3%, with 50.0% showing MRS lactate peaks. EEG epileptiform discharge burden was significantly higher than in GD/GED groups (96.7% vs. 33.3%/60.0%). Notably, among 33 patients carrying p.Arg403Cys variant, 32 developed status epilepticus. Our prior data [37] indicated that all 9 cases with RHADS EEG pattern were in the MD group (5 with p.Arg403Cys variant); among 8 patients with HHE syndrome, 7 carried p.Arg403Cys variant, suggesting HHE and RHADS may be characteristic features in this subgroup, especially among p.Arg403Cys carriers.

### 3.5 AD inheritance—GD/GED and AR inheritance—GD

The AD-GD group (*N* = 18) had a median onset age of 1 year (range: 0–30 years) and a female predominance (Male: Female = 13:5). Compared to other groups, these patients showed higher rates of peripheral neuropathy and ataxia, but lower incidence of epilepsy and encephalopathy. Other features like developmental delay and dystonia did not differ significantly. The AD-GED group (*N* = 5) involved 5 distinct variants. One patient with a p.Gln721Ter nonsense variant presented with episodic hemiplegia, astigmatism, and strabismus, with symptom improvement on L-carnitine and coenzyme Q10. The other 4 missense carriers exhibited typical *DNM1L*-related symptoms (e.g., drug-resistant epilepsy, developmental delay).

The AR-GD group (7 cases from 4 families) displayed broad phenotypic variability. A sibling pair with compound heterozygous frameshift variants died in the neonatal period due to severe hypotonia and respiratory distress, representing the most severe phenotype reported. Another sibling pair in our cohort presented with typical drug-resistant epilepsy and status epilepticus, while a third pair with compound heterozygous and homozygous missense variants had milder manifestations, limited to developmental delay and dystonia.

Prognostic outcomes varied significantly across groups. The AD-MD group had the highest mortality (32.8%, 20/61), with a median age at death of 3.3 years and median mRS score of 5. The AD-GD group showed 11.1% mortality (2/18) and median mRS of 3. Mortality in the GED and AR-GD groups was 20.0% (1/5) and 28.6% (2/7), respectively, both with median mRS of 4. Given the small sample sizes in AD-GED and AR-GD groups, all cases were stratified into MD and non-MD groups for mortality comparison. Survival analysis indicated that the MD group had twice the mortality risk of non-MD groups, with earlier age at death (*P* < 0.05) (Figures 4D, E). These findings noted a tentative difference in phenotypic severity and prognosis between different domain variants groups.

## 4 Discussion

The DRP1 protein, encoded by the *DNM1L* gene, is a highly conserved GTPase and a key regulator of mitochondrial and peroxisomal fission. DRP1 consists of an N-terminal GD, a MD, a VD, and a C-terminal GED. Upon cellular signaling, cytosolic DRP1 is recruited to the mitochondrial outer membrane, where its domains interact with specific receptors: the GD mediates membrane binding, the MD facilitates oligomerization, and the GED activates GTPase activity and stabilizes dimers, promoting DRP1 assembly into ring-like structures that drive membrane constriction (4). Pathogenic *DNM1L* variants disrupt this process, impair mitochondrial fission, and contribute to various neurological disorders.

The number of cases has been further increased relative to our previously published series (39). This study reports 18 Chinese patients carrying *DNM1L* variants, whose clinical phenotypes align with previously reported cases. Notably, 8 children exhibited a novel phenotype of HHE syndrome, expanding the clinical spectrum of the disease. HHE typically affects children aged 6 months to 4 years, often presenting with prolonged unilateral clonic seizures following fever or acute encephalopathy, followed by persistent hemiplegia, with approximately 80% eventually developing refractory focal epilepsy. Acute-phase EEG shows asymmetric discharges, and brain MRI reveals unilateral hemispheric edema progressing to hemiatrophy or global cerebral atrophy (42). All children in this group exhibited focal status epilepticus, post-ictal hemiplegia, and characteristic imaging/EEG findings. Variants were exclusively located in the MD, with 7 cases carrying the p.Arg403Cys variant. Although HHE has not been explicitly reported in prior literature, similar phenotypes associated with p.Arg403Cys have been described. Sara et al. (16) reported a 13-year-old girl who developed focal status epilepticus with fever following Measles-Mumps-Rubella (MMR) vaccination, followed by right hemiplegia and focal myoclonic seizures. Acute brain MRI showed T2/FLAIR hyperintensity in the left thalamus and putamen, and EEG revealed periodic polyspikes and slow waves over the left frontotemporal region. Jill et al. (25) described two children with similar presentations, both developing focal status epilepticus (left or right limbs) after minor metabolic stress, accompanied by contralateral hemispheric epileptiform activity and MRI abnormalities. Additionally, Danielle et al. (30) reported an adolescent female with Rasmussen's encephalitis-like symptoms, including left-sided focal status epilepticus, progressive hemiplegia, and significant asymmetric cerebral atrophy. Brain MRI and EEG indicated hemispheric pathology, and seizures persisted even after hemispherectomy. Therefore, patients presenting with febrile infection-related epilepsy syndrome (FIRES), Rasmussen's encephalitis, or HHE should be evaluated for monogenic causes such as *DNM1L* variants. These findings suggest that HHE may serve as a characteristic phenotype and prognostic indicator of severity for patients carrying MD variants, particularly p.Arg403Cys variant.

Analysis of 91 patients from this cohort and the literature revealed a median onset age of 3 years (range: 0–30 years) for *DNM1L*-related disorders. Core manifestations included psychomotor developmental delay/regression, drug-resistant epilepsy, status epilepticus, dystonia, and hemiplegia. Other common features were ataxia, nystagmus, optic atrophy, microcephaly, and peripheral neuropathy. Although rare, cardiomyopathy occurred in some patients and requires heightened clinical vigilance due to its critical nature. Most patients exhibited brain MRI abnormalities (mainly encephalopathy and atrophy) and EEG irregularities (epileptiform discharges, diffuse background slow waves). The prognosis of *DNM1L*-related disorders is poor, with a mortality rate of approximately 27.5% at last follow-up, and over half of survivors experienced severe neurological disability.

Missense mutations dominate *DNM1L* variants, with p.Arg403Cys variant being a hotspot. Variants are concentrated in the MD and GD domains. The disease primarily follows AD inheritance, mostly due to de novo heterozygous variants. Domain-specific analysis indicates that MD variants are associated with more severe phenotypes, including higher rates of refractory epilepsy, status epilepticus, encephalopathy, and worse prognosis. In contrast, GD variants more commonly present with peripheral neuropathy and ataxia, and follow a relatively milder clinical course. This highlights the importance of variant location in determining clinical expression. Furthermore, ethnic differences in variant distribution were observed: MD variants (especially p.Arg403Cys) are more frequent in Asian (particularly Chinese) populations, while GD variants are more common in Europeans. Cases involving GED variants or recessive inheritance are limited, and their phenotypic associations require validation in larger cohorts. Our study suggests a possible trend that patients with MD variants may be associated with more severe epilepsy, higher mortality and poorer prognosis, given the small sample size, this finding should be interpreted with caution and considered hypothesis-generating. This finding aids clinicians in precise diagnosis, classification, and personalized management based on variant location. It also provides clinical context for interpreting variants of uncertain significance (VUS) under ACMG guidelines in future diagnostics. This study identified eight novel pathogenic *DNM1L* variants, enriching our understanding of the genetic landscape of this disorder. Future efforts should screen for *DNM1L* variants in larger undiagnosed populations, collect clinical data and biological samples, and establish a comprehensive genotype-phenotype correlation map.

Currently, there are no targeted therapies for *DNM1L*-related mitochondrial disorders, and management remains supportive. Four children in our cohort received immunotherapies such as IVIG, corticosteroids, and tocilizumab during acute phases due to FIRES-like presentations, which partially alleviated encephalopathic symptoms but offered limited efficacy. Further research is needed to elucidate the mechanisms of epileptogenesis, clarify the role of inflammation, and explore the potential of immunotherapies. Advances in gene editing and targeted delivery technologies hold promise for future breakthroughs in treating genetic and rare diseases, including mitochondrial disorders.

Our study has several limitations. First, as a single-center retrospective analysis, phenotypic information was obtained from follow-up or medical records rather than prospective assessments. Selection bias may exist, limiting the generalizability of the results. The observed phenotypes may not cover the full variant spectrums, and some patients with clinically compatible presentations but lacking genetic testing, or those carrying VUS/LP variants with atypical phenotypes, might have been missed. Accordingly, we adopted a cautious approach in conducting genotype–phenotype correlation analyses and refrained from overinterpreting the findings. Second, subgroup comparisons stratified by protein domains, modes of inheritance, or variant types were underpowered due to the small number of cases in each group; thus, all such analyses should be regarded as exploratory and hypothesis-generating, warranting validation in larger prospective cohorts. Finally, due to young age or developmental delays, only a few patients underwent ophthalmological examinations, potentially leading to under recognition of ocular manifestations such as optic atrophy. The long-term natural history of *DNM1L*-related disorders remains poorly understood, necessitating extended follow-up data to comprehensively grasp disease progression.

This study provides the largest single-center case cohort to date. We expanded the genotypic and phenotypic spectrum of *DNM1L*-related disorders, established the association between the novel HHE phenotype and specific genotypes, and proposed its potential role as a prognostic biomarker. We also summarized common symptoms of the disease and demonstrated genotype-phenotype correlations based on protein domains and ethnic background. Continued collection of phenotypic information from rare variant carriers will help identify more features associated with specific mutations, ultimately improving disease prognosis and management.

## Data Availability

The raw data supporting the conclusions of this article will be made available by the authors, without undue reservation.
