# Supplementary material for: Clinical phenotype spectrum and prognostic analysis of DNM1L-related disorders: a single-center cohort study of 18 patients
Source: Front Neurol. 2026 Aug 19;17:1791244. doi: 10.3389/fneur.2026.1791244 (PMC13533661; doi:10.3389/fneur.2026.1791244)
Supplement: Supplementary file 1 [file Table_1.docx]

Supplementary Material

**Table S1 Demographic and clinical characteristics of the probands with *DNM1L* variants**

| **No.** | **1** | **2** | **3** | **4** | **5** | **6** | **7** | **8** | **9** |
| --- | --- | --- | --- | --- | --- | --- | --- | --- | --- |
| **Sex** | M | M | M | M | M | F | F | F | M |
| **Age of onset（y）** | 3.4 | 0.1 | 5.0 | 2.5 | 0.6 | 3.0 | 4.8 | 5.8 | 5.0 |
| **Family History** | Negative | Negative | Negative | Negative | Negative | Negative | Negative | Negative | Negative |
| **Initial**  **symptoms** | Cyclic vomit | Feeding difficulties | Seizure | Developmental delay | Motor retardation | ASD | Seizure | Seizure | Seizure |
| **Age of seizure（y）** | 4.3 | 0.2 | 5.0 | 8.8 | **-** | 6.3 | 4.8 | 5.8 | 5.0 |
| **Seizure type** | | | | | | | | | |
| Focal | **+** | **-** | **+** | **+** | **-** | **+** | **+** | **+** | **+** |
| Generalized | **-** | **+** | **+** | **-** | **-** | **-** | **-** | **-** | **-** |
| **Frequency**  **of seizure** | Daily | Daily | Monthly-Yearly | Monthly | **-** | Monthly | Daily | Monthly | Daily |
| **SRSE** | **+** | **-** | **+** | **-** | **-** | **+** | **+** | **+** | **+** |
| **ASMs** | 8 | 5 | 8 | 2 | **-** | N/A | 5 | 6 | 7 |
| **Anesthetic**  **drug therapy** | Chloral  hydrate, Midazolam, Propofol | **-** | Chloral hydrate, Midazolam, Propofol | **-** | **-** | **-** | Midazolam, Propofol | Diazepam, Dex | Midazolam, Propofol |
| **EEG discharge** | | | | | | | | | |
| Circumscribed | **+** | **-** | **+** | **-** | **-** | **+** | **+** | **+** | **+** |
| Widespread | **+** | **+** | **+** | **+** | **-** | **-** | **-** | **-** | **-** |
| **MRI Abnormity** | | | | | | | | | |
| Yes | Cerebral atrophy, encephalomalacia,  Gliosis | **-** | Atypical tuberous sclerosis, Periventricular cysts | **-** | **-** | N/A | Extensive cerebral atrophy, | Cerebral atrophy | Cerebral atrophy |
| No | **-** | **+** | **-** | **+** | **+** | N/A | **-** | **-** | **-** |
| **Neurodevelopment** | | | | | | | | | |
| Before onset  (retardation) | Mild | Moderate | Mild | Mild | Mild | Mild | Mild | **-** | - |
| After onset  (regression) | **+** | **-** | **-** | **-** | **-** | **-** | **-** | **-** | **-** |
| **Other Neurological signs** | | | | | | | | | |
| Dystonia | **+** | **+** | **-** | **+** | **-** | **-** | **+** | **-** | **+** |
| Ataxia | **-** | N/A | N/A | **-** | **-** | **-** | **-** | **+** | **-** |
| Hemiparesis | **+** | N/A | N/A | **-** | **-** | **-** | **-** | **+** | **-** |
| Optic Atrophy | N/A | N/A | N/A | N/A | **-** | N/A | **-** | N/A | N/A |
| Nystagmus | **-** | N/A | N/A | **-** | **-** | **-** | **-** | **-** | **+** |
| Others | Positive  ankle clonus | Dysphagia | Microcephaly | **-** | **-** | Dysarthria | Positive ankle clonus, Dyskinesia | **-** | Dysarthria |
| **Hyperlacticaemia** | **-** | **-** | **-** | **+** | **-** | **-** | **+** | **-** | **-** |
| **Other systemic symptoms** | **-** | **-** | Rickets | Hyperemia bulbar | **-** | **-** | **-** | **-** | - |
| **Other Treatments** | Cocktail  therapy, GC、IVIG、Bezafibrate | Cocktail  therapy | Cocktail therapy,  GC | L-carnitine | - | **-** | Cocktail therapy | Cocktail  therapy,  GC | Cocktail  therapy |
| **Other laboratory indicators** | **-** | Atrial septal defect, Ventricular hypertrophy | Elevated creatine  kinase | **-** | **-** | **-** | **-** | **-** | **-** |
| **Follow-up（m）** | 16 | 5 | 28 | 14 | 24 | 38 | 17 | 19 | 18 |
| **Outcome（Died）** | N | Y | N | N | N | N | N | N | N |
| **Rankin Score** | 5 | 6 | 5 | 3 | 2 | 4 | 5 | 5 | 4 |

**Continued- Table S1 Demographic and clinical characteristics of the individuals with *DNM1L* variants**

| **No.** | **10** | **11** | **12** | **13** | **14** | **15** | **16** | **17** | **18** |
| --- | --- | --- | --- | --- | --- | --- | --- | --- | --- |
| **Sex** | M | M | F | F | F | F | M | F | M |
| **Age of onset（y）** | 0.3 | 0.3 | 10.8 | 4.3 | 3.0 | 5.6 | 7.1 | 0.3 | 3.5 |
| **Family History** | Negative | Negative | Negative | Negative | Negative | Negative | Negative | Negative | Negative |
| **Initial symptoms** | Developmental delay | Developmental delay | Myasthenia | Seizure | Developmental delay | Seizure | Seizure | Developmental delay | Seizure |
| **Age of seizure（y）** | 3.2 | 4.2 | 11.5 | 4.3 | 6.5 | 5.6 | 7.1 | - | 3.5 |
| **Seizure type** | | | | | | | | | |
| Focal | **+** | **+** | **+** | **+** | **+** | **+** | **+** | **-** | **+** |
| Generalized | **-** | **-** | **-** | **-** | **+** | **-** | **+** | **-** | **-** |
| **Frequency of seizure** | Daily | Daily | Weekly | Daily | Daily | Daily | Daily | - | Daily |
| **SRSE** | **+** | **+** | **-** | **+** | **+** | **+** | **+** | **-** | **+** |
| **ASMs** | 3 | 4 | 1 | 6 | 6 | 8 | 8 | - | 7 |
| **Anesthetic**  **drug therapy** | Diazepam | Chloral hydrate, Midazolam | **-** | Chloral hydrate  Midazolam  Dex | Chloral  hydrate Midazolam  Dex | Midazolam | Diazepam Midazolam Propofol | **-** | Midazolam, Propofol |
| **EEG discharge** | | | | | | | | | |
| Circumscribed | **+** | **-** | **+** | **+** | **+** | **+** | **-** | **-** | **+** |
| Widespread | **+** | **+** | **+** | **+** | **+** | **+** | **+** | **-** | **-** |
| **MRI Abnormity** | | | | | | | | | |
| Yes | Extensive edema of the right cerebral cortex, abnormal signals in the basal ganglia | Extensive edema of the left cerebral cortex | **-** | Cerebral atrophy | Cerebral atrophy | Cerebral atrophy | Bilateral basal ganglia, thalamus, and cortical areas with multiple abnormal signals | Delayed myelination | Cerebral atrophy |
| No | **-** | **-** | **-** | **-** | **-** | **-** | **-** | **-** | **-** |
| **Neurodevelopment** | | | | | | | | | |
| Before onset  (retardation) | Mild | Mild | **-** | - | Mild | **-** | **-** | Moderate | **-** |
| After onset  (regression) | **-** | **-** | **-** | **-** | **+** | **+** | **+** | **-** | **+** |
| **Other Neurological signs** | | | | | | | | | |
| Dystonia | **+** | **+** | **+** | **+** | **-** | **+** | **+** | **-** | **+** |
| Ataxia | **-** | **-** | **-** | **-** | **-** | N/A | **-** | **-** | **-** |
| Hemiparesis | **+** | **+** | **-** | **-** | **-** | **-** | **-** | **-** | **-** |
| Optic Atrophy | N/A | N/A | N/A | **+** | N/A | N/A | N/A | **-** | N/A |
| Nystagmus | **-** | **-** | **-** | **-** | **-** | **-** | **-** | **-** | **+** |
| Others | Dyskinesia | Dyskinesia | Syringomyelia | Positive ankle clonus, Active tendon reflexes | Spina bifida occulta, Dysarthria | **-** | Positive Babinski sign | - | Dysarthria |
| **Hyperlacticaemia** | **-** | **-** | **-** | **+** | **-** | **-** | **-** | **-** | **-** |
| **Other systemic symptoms** | **-** | Biliary atresha | **-** | **-** | **-** | **-** | **-** | **-** | - |
| **Other Treatments** | Cocktail therapy, GC, IVIG, Tocilizumab | Cocktail therapy, GC, Tacrolimus, Tocilizumab, IVIG | **-** | Cocktail therapy | Cocktail therapy, GC, IVIG | Cocktail therapy, GC, IVIG, TocilizumabBezafibrate | Cocktail therapy, GC, IVIG | Cocktail  therapy | Cocktail  therapy |
| **Other laboratory indicators** | Elevated  IL-6 | **-** | Multiple peripheral damage in EMG | **-** | **-** | **-** | HA, Elevated  IL-6 | **-** | **-** |
| **Follow-up（m）** | 22 | 4 | 4 | 34 | 34 | 7 | 8 | 2 | 18 |
| **Outcome（Died）** | N | N | N | N | N | N | N | N | N |
| **Rankin Scale** | 5 | 5 | 3 | 4 | 5 | 5 | 4 | 5 | 4 |

Abbreviation: M:Male; F: Female; y: year; m: month; SRSE: Super refractory status epilepticus; ASMs: Antiseizure medications; EEG: Electrocorticogram; MRI: Magnetic resonance imaging; Y: Yes; N: No; N/A: Not Available; ASD: Autism spectrum disorders; IVIG: Intravenous Immunoglobulin; Glucocorticoids: GC; EMG: Electromyography; APB: Atrial premature beats; HA: Hyperammonemia; Dex: Dexmedetomidine; IL-6: Interleukin-6.

**Table S3 MRI imaging abnormalities of *DNM1L* patients**

| **Case** | **Age of scanning（years）** | **MRI sequence** | **Main abnormalities** | **The abnormalities progress over time** |
| --- | --- | --- | --- | --- |
| 1 | 4.5 | TIWI, T2WI, T2-FLAIR | Cerebral atrophy, encephalomalacia, Gliosis | Yes |
| 2 | 7.4 | TIWI, T2WI, T2-FLAIR, DWI | Atypical tuberous sclerosis, Periventricular cysts | No |
| 7 | 4.7 | TIWI, T2WI, T2-FLAIR, DWI | Extensive edema of the left frontal-parietal lobe cerebral cortex, abnormal signals in the left thalamus | Yes |
| 8 | 5.8 | TIWI, T2WI, T2-FLAIR | cerebral atrophy | Yes |
| 9 | 9.8 | TIWI, T2WI, T2-FLAIR | cerebral atrophy | Yes |
| 10 | 3.2 | TIWI, T2WI, T2-FLAIR, DWI | Extensive edema of the right cerebral cortex, abnormal signals in the basal ganglia | Yes |
| 11 | 3.9 | TIWI, T2WI, T2-FLAIR, DWI | Extensive edema of the left cerebral cortex | Yes |
| 12 | 11.1 | TIWI, T2WI, T2-FLAIR | Lumbar syringomyelia | No |
| 13 | 4.9 | TIWI, T2WI, T2-FLAIR | Cerebral atrophy | Yes |
| 14 | 6.5 | TIWI, T2WI, T2-FLAIR, DWI | Cerebral atrophy | Yes |
| 15 | 5.5 | TIWI, T2WI, T2-FLAIR, DWI | Cerebral atrophy | Yes |
| 16 | 7.1 | TIWI, T2WI, T2-FLAIR, DWI | Bilateral basal ganglia, thalamus, and cortical areas with multiple abnormal signals | Yes |
| 17 | 1.8 | TIWI, T2WI, T2-FLAIR | Delayed myelination | No |
| 18 | 7.2 | TIWI, T2WI, T2-FLAIR | Cerebral atrophy | Yes |
|  |  |  |  |  |

Abbreviation: MRI: Magnetic resonance imaging; T1WI: T1-weighted imaging; T2WI: T2-weighted imaging; T2-FLAIR: Fluid-attenuated inversion-recovery sequency; DWI: Diffusion-weighted imaging.
